# MiR-34c downregulation leads to SOX4 overexpression and cisplatin resistance in nasopharyngeal carcinoma

**DOI:** 10.1186/s12885-020-07081-z

**Published:** 2020-06-26

**Authors:** Pierre-Antoine Bissey, Mona Teng, Jacqueline H. Law, Wei Shi, Jeff P. Bruce, Valentin Petit, Sai W. Tsao, Kenneth W. Yip, Fei-Fei Liu

**Affiliations:** 1 Princess Margaret Cancer Centre, University Health Network, Toronto, Canada; 2 Department of Medical Biophysics, University of Toronto, Toronto, Canada; 3 LabEx DEVweCAN, Université de Lyon, F-69000 Lyon, France; 4 School of Biomedical Sciences, Li Ka Shing Faculty of Medicine, University of Hong Kong, Hong Kong, China; 5 Radiation Medicine Program, Princess Margaret Cancer Centre, University Health Network, 610 University Avenue, Toronto, Ontario M5G 2M9 Canada; 6 Department of Radiation Oncology, University of Toronto, Toronto, Canada

**Keywords:** miR-34c, SOX4, TGFβ1, EMT, Nasopharyngeal cancer, Cisplatin

## Abstract

**Background:**

A major cause of disease-related death in nasopharyngeal carcinoma (NPC) is the development of distant metastasis (DM) despite combination chemoradiotherapy treatment. We previously identified and validated a four microRNA (miRNA) signature that is prognostic for DM. In this study, characterization of a key component of this signature, miR-34c, revealed its role in chemotherapy resistance.

**Methods:**

Two hundred forty-six NPC patient biopsy samples were subject to comprehensive miRNA profiling and immunohistochemistry (IHC). Two human normal nasopharyngeal cell lines (immortalized; NP69 and NP460), as well as the NPC cell line C666–1, were used for miR-34c gain-of-function and loss-of-function experiments. Signaling pathways were assessed using quantitative real-time PCR (qRT-PCR) and Western blot. Cell viability was measured using the ATPlite assay.

**Results:**

MiR-34c was downregulated in NPC patient samples, and confirmed in vitro to directly target SOX4, a master regulator of epithelial-to-mesenchymal transition (EMT). MiR-34c downregulation triggered EMT-representative changes in NP69 and NP460 whereby Snail, ZEB1, CDH2, and SOX2 were upregulated, while Claudin-1 and CDH1 were downregulated. Phenotypically, inhibition of miR-34c led to cisplatin resistance, whereas miR-34c over-expression sensitized NPC cells to cisplatin. TGFβ1 decreased miR-34c and increased SOX4 expression in vitro. The TGFβ receptor 1 inhibitor SB431542 reduced SOX4 expression and increased cisplatin sensitivity. Finally, IHC revealed that lower SOX4 expression was associated with improved overall survival in chemotherapy-treated NPC patients.

**Conclusion:**

miR-34c is downregulated in NPC. Repression of miR-34c was shown to increase SOX4 expression, which leads to cisplatin resistance, while TGFβ1 was found to repress miR-34c expression. Taken together, our study demonstrates that inhibition of the TGFβ1 pathway could be a strategy to restore cisplatin sensitivity in NPC.

## Background

Nasopharyngeal carcinoma (NPC) patients presenting with locally advanced disease have a very modest overall survival (OS) rate of approximately 65% after 5 years [1–3]. Despite the use of intensity-modulated radiation therapy for this Epstein-Barr virus (EBV)-associated malignancy, 20–30% of NPC patients will still succumb to distant metastasis (DM) [4]. Therapeutic options for such NPC patients are limited, and a primary clinical challenge is resistance to chemoradiation [5]. Concurrent chemotherapy (cisplatin/5-fluorouracil) with radiation therapy (RT) modestly improves OS, but can cause significant toxicity and death [4, 6–10].

Our group previously completed a global miRNA NPC patient sample profiling, discovering and validating a four-microRNA (miRNA) prognostic signature associated with risk for DM (low miR-34c, low miR-140, high miR-154, and high miR-449b) [11]. A subsequent study demonstrated that elevated levels of miR-449b were significantly associated with poor OS in patients receiving concurrent chemoradiotherapy [12]. MiR-449b overexpression in NPC was found to decrease transforming growth factor beta-induced (TGFBI), leading to an increase in transforming growth factor beta 1 (TGFβ1), TGFβ pathway activation, and cisplatin resistance [12].

TGFβ1 is a secreted protein involved in the regulation of many cellular mechanisms, such as metastasis formation, chemoresistance, epithelial-to-mesenchymal transition (EMT) [13, 14], and more recently, miRNA expression [15, 16]. This latter process occurs via TGFβ1-mediated Smad activation whereby Smads bind to miRNA promoter regions that contain Smad-binding elements, as well as the Drosha complex [17]. Conversely, numerous miRNAs have been shown to negatively regulate TGFβ pathways [18].

TGFβ1 mediates the overexpression of SOX4, a member of the SOX (SRY-related HMG-box) family of transcription factors, which are known to be involved in developmental pathologies and cancer [19–22]. SOX4 dysregulation is involved in a myriad of cellular phenomena, such as the cell cycle, apoptosis, response to chemoradiation, metastasis development, and EMT [19, 23–27]. It is highly expressed in prostate [28], glioma [29], gastric [30], and breast cancers [27, 31], and its elevated expression, in turn has been associated with worse survival in prostate [32], gastric [30, 33], and colon cancers [34], as well as NPC [35]. The opposite however, has also been observed in several other malignancies, suggesting that the involvement of SOX4 may be context-dependent [36, 37].

Another component of the four-miRNA DM signature is miR-34c, which was only compared to other miRNAs within NPC, but not assessed in healthy individuals [11]. Other groups have shown miR-34c downregulation in NPC compared to normal tissue [38, 39], which has also been demonstrated in several other cancers [40–43]. MiR-34c is a member of the miR-34 family, which is composed of three pro-apoptotic members: miR-34a, miR-34b, and miR-34c, all of which have been described as transcriptional targets of p53 [44]. MiR-34a is located on chromosome 1p36, whereas miR-34b/c are located on chromosome 11q23 [45]. While extensive research has been conducted on miR-34a [46], identifying its role in chemosensitivity [47, 48], prevention of metastasis formation [49–52], and reverting EMT [53, 54], there is a paucity of information regarding miR-34c.

In this current study, the biological mechanisms and effects of miR-34c downregulation were investigated. The data suggest that this downregulation is caused by TGFβ1, which leads to SOX4 disinhibition, which in turn promotes EMT and cisplatin resistance in NPC – two features that contribute to the formation of DM.

## Methods

### Patient samples

In compliance with the Institutional Research Ethics Board at the University Health Network (UHN), all patients provided written consent for the use of their tissues in this study. Diagnostic formalin-fixed paraffin-embedded (FFPE) blocks were obtained from NPC patients (*n* = 246) treated at the Princess Margaret Cancer Center (PMCC) between 1993 to 2009, as previously described [11]. FFPE tissues from patients who underwent quadroscopy and were not diagnosed with NPC (*n* = 17) were used as normal nasopharyngeal epithelial tissues.

### NanoString analysis

RNA was isolated using the Recover All Total Nucleic Acid Isolation Kit for FFPE (Ambion, Austin, TX, USA). Total RNA (200 ng) was assayed using the nCounter Human miRNA Assay v1.0 (Nanostring; 734 unique human and viral miRNAs). Please note that this experiment was also used for a previous study. Full analyses and protocols can be found in Bruce et al. [11].

### Cell culture

The EBV-positive NPC cell line C666–1, the non-tumorigenic human nasopharyngeal cell lines NP69 (SV40-immortalized) and NP460 (hTert-immortalized), and HEK 293 T cells were cultured as previously described [12]. NP69 and NP460 cell lines were generated by SW Tsao’s group [55, 56] and served as “normal” cells throughout this study. Every new batch of cells underwent mycoplasma testing and STR analyses [12]. C666–1, NP69 and NP460 cells were used for gain- and loss-of-function assays; HEK 293 T (ATCC CRL-32 L) cells were used for lentiviral generation and luciferase assays.

### Compound treatments

SB431542 (#S1067, SelleckChem, Houston, TX, USA), a TGFβ receptor I (TGFβR1, also known as ALK5) inhibitor, was used as indicated. Human TGFβ1 (#8915; Cell Signaling, Danvers, MA, USA) was used where indicated after overnight starvation of cells in Minimum Essential Media (MEM) supplemented with 0.5% FBS.

### Transfection

Polyplus-transfection JetPRIME (Graffenstaden, France) was used for transfection of C666–1, NP69, NP460, and HEK 293 T cells, according to manufacturer’s specifications. C666–1, NP69, and NP460 cells were transfected with pre-miR-34c or pre-miR negative control (20 nM and 50 nM, Ambion, Austin, TX, USA).

### Lentiviral transduction

Lentiviral transduction was used to generate stable cell lines as previously described [12]. pLV-miRNA-34c (Biosettia, San Diego, CA, USA), pLV-miR-34c-lockers (Biosettia, San Diego, CA, USA), and their respective control vectors were used. All stable cell lines were generated for the purpose of this work.

### Quantitative real-time PCR (qRT-PCR)

The Total RNA Purification Kit (Norgen Biotek, Thorold, ON, Canada) was used for both mRNA and miRNA isolation. Reverse-transcription of total RNA (1 μg) was performed using the iScript cDNA Synthesis Kit (BioRad, Hercules, CA, USA). qRT-PCR was performed using SYBR Green (Roche, Basel, Switzerland) and the primers are listed in Table 1. mRNA expression was normalized to the average expression of two housekeeping genes (β-actin and GAPDH, as in [12]) and melting curves were generated for each experiment. MiRNA levels were assessed using the TaqMan MicroRNA Assay, and processed according to manufacturer’s instructions (Applied Biosystem, Foster City, CA, USA). RNU44 and RNU48 were used to normalize miR-34c expression [57, 58]. Relative expression was calculated using the 2^-ΔΔCt^ method [59].

**Table 1:** Oligonucleotides used for qRT-PCR

Gene	Forward Primer (5′ to 3′)	Reverse Primer (5′ to 3′)
β-actin	AGAGCTACGAGCTGCCTGAC	AGCACTGTGTTGGCGTACAG
ARID5A	ACCAGATGATGCCAGGAAAG	GAGCTTCTTTTTGGCCAGTG
BAX	GGGTGGTTGCCCTTTTCTACT	CCCGGAGGAAGTCCAGTGTC
BIK	AAGACCCCTCTCCAGAGACAT	CAAGAACCTCCATGGTCGGG
CCL22	ACTGCACTCCTGGTTGTCCT	CGGCACAGATCTCCTTATCC
GAPDH	TGTTGCCATCAATGACCCCTT	CTCCACGACGTACTCAGCG
LITAF	TCGGTTCCAGGACCTTACCA	GGAGGATTCATGCCCTTCCC
MARCKS	CCCAGTTCTCCAAGACCGC	CTGTCCGTTCGCTTTGGAAG
MR1	GACTCGCACCCTATCACCAC	CGAGGTTCTCTGCCATCCAT
NFKBIA	GAAGTGATCCGCCAGGTGAA	CTGCTCACAGGCAAGGTGTA
NOTCH1	TCCACCAGTTTGAATGGTCA	AGCTCATCATCTGGGACAGG
PDE4B	GGAAAAATCCCAGGTTGGTT	AGTGGTGGTGAGGGACTTTG
PML	GGCAGAGGAACGCGTTGTGGT	GGCTGGATGACCACGCGGAA
RANGAP1	TCAAGAGCTCAGCCTGCTTC	TTCCGGTGACATTCGGTCAG
RBM4	CTTGAGGTGGGATGTGTGTG	GCAGGAGAGGAAAGGAAAGG
RNF24	TGAGTTGGGGATTTGTCCAT	TACTTTGCGAACTTCCAGCC
SOX2	GCTACAGCATGATGCAGGACCA	TCTGCGAGCTGGTCATGGAGTT
SOX4	CCAAATCTTTTGGGGACTTTT	CTGGCCCCTCAACTCCTC
TGIF2	TGAAGATCCTCCGGGACTGG	CAGCACTGACAGGTTGGTCT
TRIO	AGCACACCTGGACCTAAAGC	GCACTCCAACACTCCACGTA

### Western blot

Immunoprecipitation buffer (150 mM NaCl, 5 mM EDTA, 50 mM Hepes pH 7.6, 1–2% Nonidet P-40; with protease inhibitor cocktail, Roche), was used for protein extraction. Electrophoresis was performed with Bolt 4–20% Gels (Life Technologies, Carlsbad, CA, USA).

The Epithelial-Mesenchymal Transition Antibody Sampler Kit (Cell Signaling; #9782; 1/1000 each), anti-TGFβ1 (Cell Signaling; #3711; 1/1000), and anti-β-actin (Sigma: 1/5000) antibodies were used. The SuperSignal West Femto ECL (Pierce, #34095, Thermo Scientific, Waltham, MA, USA) was used for ZEB1, CDH1 and ZO-1 detection. Pierce ECL (#32209) was used to detect all other proteins.

### RNA sequencing (RNA-Seq) and data analysis

RNA from our cohort of FFPE samples was isolated (200 ng/sample), processed (Ribo-Zero Gold rRNA Removal Kit (Illumina, San Diego, CA, USA)), and sequenced as previously described (as in the NanoString section of [11]). A subset of these samples (*n* = 53) was processed for RNA-seq. Library preparation was performed using the TruSeq Stranded Total RNA Sample Prep Kit (Illumina, San Diego, CA, USA). Sequencing was conducted on the Illumina HiSeq 2000 to > 100 million paired-end 100 bp reads. STAR (v2.4.2a) was used to align the reads [60], and RSEM (v1.2.21) was used to summarize expression values [61].

### Luciferase reporter assay for MiR-34c/SOX4 target activity

MiR-34c was predicted to target the wild-type (WT) 3′-untranslated region (3’UTR) of SOX4 in silico. This region was inserted into the pMIR-REPORT vector (Ambion). JetPRIME was used to reverse transfect HEK 293 T cells with pre-miR-control or pre-miR-34c. Twenty-four hours later, JetPRIME was used to co-transfect pRL-SV Renilla vector (Promega, Madison, WI, USA) with either pMIR-SOX4 3’UTR WT (CTAGTGCTCAGCTCAAGTTCACTGCCTGTCAGAT) or pMIR-SOX4 3’UTR Mutant (CTAGTGCTCAGCTCAAGTTTCTGTAAAGTCAGAT). The Dual-Luciferase Reporter Assay (Promega) was used to measure luciferase activity 24 h post-transfection.

### Cell viability assays

Stable cell lines generated from C666–1, NP69 and NP460 cells were seeded in 96-well plates (2000 cells/well). After 1 day, they were exposed to decreasing concentrations of cisplatin (CDDP) for 72 h as indicated in the figures. Dose-response curves for cisplatin were determined through treatment using two-fold serial dilutions starting from 12.5 μg/mL (which induced ~ 90% cell death in NP69/NP460 cells after 72 h of treatment). Cell viability was assessed using the ATPlite 1 Step Luminescence Assay System (PerkinElmer, Waltham, MA, USA).

### Immunohistochemistry (IHC)

Sections from FFPE blocks were subject to IHC using microwave antigen retrieval. Citric acid (0.01 M, pH 6.0) and the LSAB+ System-HRP (Dako, Les Ulis, France) were used. Rabbit polyclonal anti-SOX4 (PA5–41442, lot#SB2344261A, Invitrogen: 1/40) antibody was used, but omitted for negative control staining. Positive nuclear SOX4 localization was detected by light microscopy. The percentage of positive tumour cells was quantified by evaluating a total of at least 300 tumour cells from the three most densely staining fields (magnification 400×). A final score was calculated as the product of the percentage of positive tumour cells and staining intensity (0 = negative; 1 = weak; 2 = moderate; 3 = strong) as previously described [62]. No samples had an intensity score of 3. All scoring was performed blinded to any knowledge of clinical or pathological parameters. Each section was scored at least twice.

### Statistical analyses

All experiments were performed at least three times. In order to maintain independence between replicates, new frozen batches of cells were used each time. Data are presented as the mean ± SEM. GraphPad Prism (GraphPad Software, San Diego, CA, USA) was used for statistical analyses. Intergroup statistical significance was determined using the ANOVA test, with the Bonferroni post-test (if applicable), or the Mann-Whitney *U* test (socscistatistics.com).

## Results

### MiR-34c is downregulated by TGFβ1

**Fig. 1:**
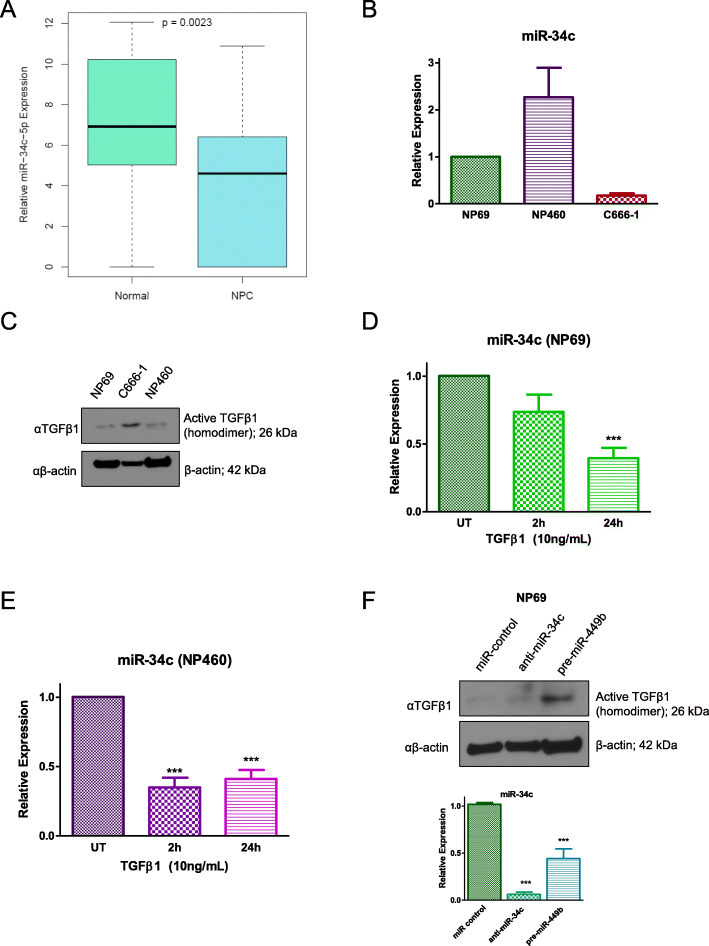
MiR-34c is under-expressed in NPC and downregulated by TGFβ1. **a** Relative miR-34c expression in normal patients (not diagnosed with NPC) vs. NPC patients (using data from Bruce et al., 2014 [11]). **b** Relative expression (qRT-PCR) of miR-34c in NP69, NP460, and C666–1 cell lines, normalized to NP69 cells. **c** Whole cell lysate (WCL) Western blotting (WB) of NP69, C666–1, and NP460 cells using anti-TGFβ1 antibody (αTGFβ1), with anti-β-actin (αβ-actin) as the loading control. Full-length blots are presented in Additional file 5: Figure S5. (D and E) Relative miR-34c expression assessed by qRT-PCR after treatment with 10 ng/mL of recombinant TGFβ1 in NP69 (**d**) and NP460 (**e**) cells. UT = untreated. **f** WB performed on WCL of stably transfected NP69-miR-control, NP69-anti-miR-34c, and NP69-pre-miR-449b cells using anti-TGFβ1 antibody, with anti-β-actin (αβ-actin) as the loading control (top); corresponding relative miR-34c expression assessed by qRT-PCR (bottom). Full-length blots are presented in Additional file 5: Figure S5. The data are represented as the mean ± SEM of at least three independent experiments. *** *P* < 0.001

In order to investigate the role of miR-34c downregulation in the validated prognostic signature for NPC DM [11], we first confirmed that miR-34c expression was significantly reduced in NPC diagnostic FFPE samples compared to normal nasopharyngeal tissues using previously generated NanoString data [11] (Fig. 1a). Cell line models were then assessed for miR-34c expression. EBV-positive NPC cell line C666–1 exhibited significantly lower levels of miR-34c compared to the two normal (immortalized) nasopharyngeal cell lines NP69 and NP460 (Fig. 1b), consistent with clinical observations.

We had previously demonstrated that miR-449b overexpression, another component of the validated prognostic DM signature [11], led to TGFBI mRNA degradation with subsequent TGFβ1 accumulation [12]. Given that TGFβ1 plays an important role in NPC progression [53, 63–68] and in the regulation of miRNAs, particularly miR-34a [52], we sought to measure TGFβ1 in these cell lines. Indeed, C666–1 cells (which have high miR-449b expression [12]) expressed higher levels of active TGFβ1 compared to either NP69 or NP460 cells (both of which have lower miR-449b expression [12]) (Fig. 1c). We therefore hypothesized that TGFβ1 could be regulating miR-34c in these cells. Treatment with recombinant TGFβ1 significantly reduced miR-34c expression in both NP69 and NP460 cells (Fig. 1d and e). Conversely, a TGFβ receptor 1 (TGFBR1) inhibitor (SB431542) increased miR-34c expression in C666–1 cells (Additional file 1: Figure S1A).

In order to confirm the association between increased miR-449b, increased TGFβ1, and decreased miR34c, NP69 cells stably expressing pre-miR-449b were compared to NP69 cells stably expressing miR-control or anti-miR-34c. NP69-pre-miR-449b cells expressed higher levels of active TGFβ1 protein compared to NP69-miR-control or NP69-anti-miR-34c cells (Fig. 1f, top); associated with a correspondingly lower expression of miR-34c compared to NP69-miR-control (Fig. 1f, bottom). Taken together, these data support the hypothesis that TGFβ1 decreases miR-34c expression, although the mechanism of regulation remains unknown.

### MiR-34c directly downregulates SOX4

**Fig. 2:**
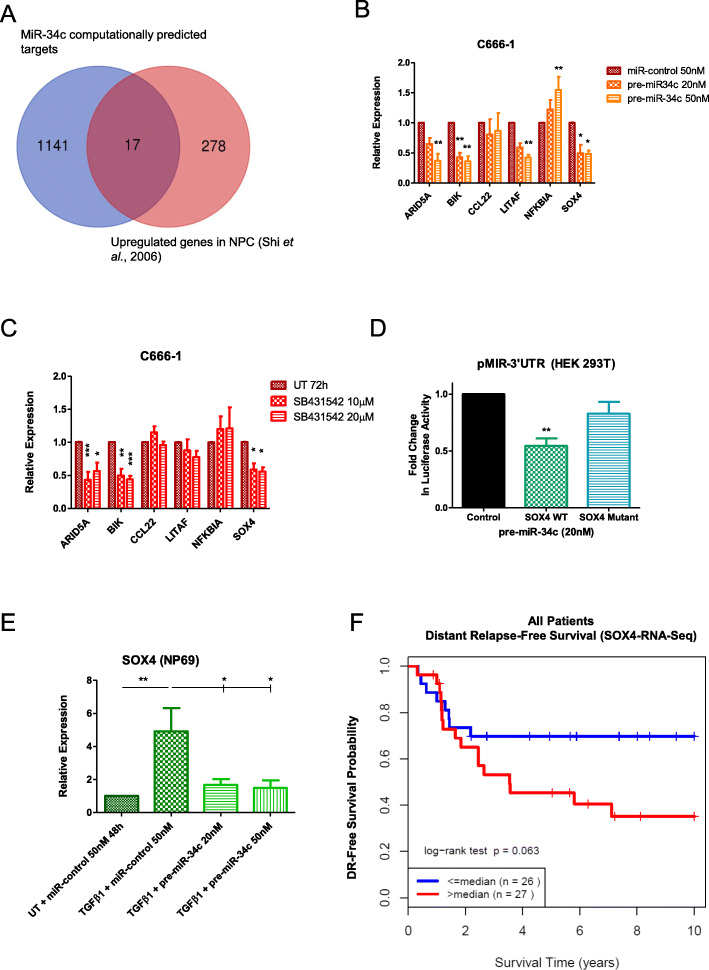
MiR-34c inhibits SOX4 expression. **a** Evaluation of miR-34c targets: the Venn diagram was generated by combining miRWalk-predicted miR-34c targets and the upregulated NPC genes from Shi et al., 2006 [69] using the online tool at www.bioinformatics.psb.ugent.be/webtools/Venn. **b** and **c** qRT-PCR of genes highly expressed in C666–1 cells compared to NP69/NP460 cells. **b** C666–1 cells were transiently transfected with pre-miR-34c (20 or 50 nM) for 72 h. **c** C666–1 cells were treated with SB431542 (10 or 20 μM) for 72 h. **d** Relative luciferase activity after transient transfection with pre-miR-34c (20 nM) for 48 h, followed by co-transfection with Renilla plasmid (100 ng) and either pMIR-SOX4 3’UTR Wildtype (WT) (150 ng) or pMIR-SOX4 3’UTR Mutant (150 ng) for 24 h. **e** qRT-PCR for SOX4 in NP69 cells transiently transfected with miR-control (50 nM), or pre-miR-34c (20 or 50 nM) for 72 h; 8 h after transfection, the media was changed to MEM 0.5% FBS; the following morning, cells were treated with recombinant TGFβ1 (10 ng/mL) for 48 h. **f** Kaplan-Meier plot of DRFS for NPC patients (*n* = 53) dichotomized based on low (<median) vs. high (>median) SOX4 mRNA expression (median follow-up time = 6 years). The data are represented as the mean ± SEM of at least three independent experiments. * *P* < 0.05; ** *P* < 0.01; *** *P* < 0.001

In order to identify miR-34c target candidates, 17 genes at the intersection between computationally predicted targets and genes upregulated in patient NPC samples [69] were examined (Fig. 2a). Using qRT-PCR, 6 of the 17 genes were observed to be upregulated in C666–1 (low miR-34c) compared to NP69 and NP460 cells (high miR-34c) (Additional file 1: Figure S1B and C). These genes were then assessed for response to transient miR-34c overexpression (pre-miR-34c transfection) (Fig. 2b for the 6 genes; Additional file 2: Figure S2A for the other 11 genes), and TGFβ pathway inhibition using SB431542 (a TGFBR1 inhibitor, which also upregulates miR-34c) (Fig. 2c for the 6 genes; Additional file 2: Figure S2B for the remaining 11 genes) in C666–1 cells. As can be seen in Fig. 2b and c, elevated miR-34c conditions consistently and significantly downregulated ARID5A, BIK, and SOX4. Interestingly, BAX and PML were consistently and significantly upregulated (Additional file 2: Figure S2A and B), suggesting that they are not direct targets of miR-34c, but possibly further downstream or altered via a more complex mechanism.

The expression of the potential miR-34c targets was then determined through qRT-PCR on NP69 and NP460 cells transiently transfected with pre-miR-34c (Additional file 2: Figure S2C and D), as well as on NP69, NP460, and C666–1 cells stably expressing pre-miR-34c and anti-miR-34c (Additional file 2: Figure S2E, F, G, H, I and J). Together, these data show that only SOX4 was both significantly and inversely related to miR-34c in all tested cell line models. SOX4 is potentially important in the tumorigenesis of a number of different cancers (reviewed in [70]), including NPC [35, 71]. It is also known to be regulated by TGFβ1 [19], although its relationship with miR-34c remains to be investigated. Thus, we proceeded to interrogate the relationship between the TGFβ pathway, miR-34c, and SOX4.

First, miR-34c–mediated direct inhibition of SOX4 expression was confirmed using a luciferase reporter assay (Fig. 2d). The data were corroborated in NP69 cells, wherein TGFβ1 treatment significantly increased SOX4 expression, which was abrogated with miR-34c overexpression (Fig. 2e). Furthermore, RNA-seq performed on 53 diagnostic NPC biopsy samples revealed that patients with higher than median SOX4 transcript levels experienced a lower 10-year distant relapse-free survival (DRFS) compared to those with lower levels (*p* = 0.063) (Fig. 2f). Taken together, these data suggest that elevated TGFβ1 (via miR-449b upregulation (Fig. 1f) and consequent TGFBI degradation [12]) may lead to the downregulation of miR-34c, which directly upregulates SOX4 overexpression, possibly leading to an inferior 10-year DRFS, as seen in this dataset.

### MiR-34c regulates the SOX2-EMT Axis

**Fig. 3:**
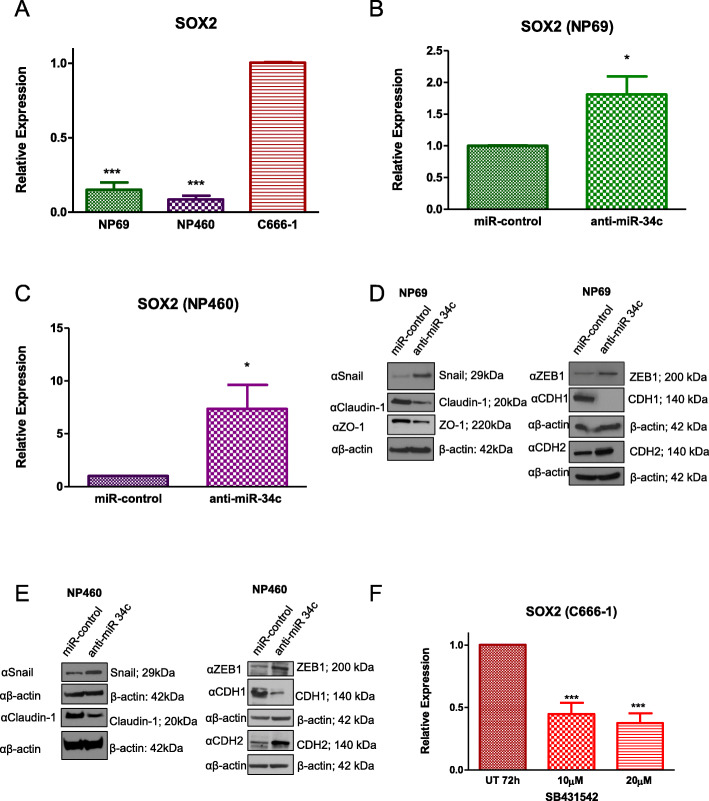
MiR-34c regulates the SOX2-EMT axis in NPC cell lines. **a**, **b** and **c** Relative expression (qRT-PCR) of SOX2: **a** in NP69, NP460 and, C666–1 cell lines, normalized to C666–1 cells; **b** in NP69-anti-miR-34c stable cells and their control; **c** in NP460-anti-miR-34c stable cells and their control. **d** and **e** Western blots (WBs) were performed using anti-Snail (αSnail), anti-Claudin-1 (αClaudin-1), anti-ZO-1 (αZO-1), anti-ZEB1 (αZEB1), anti-CDH1 (αCDH1), anti-CDH2 (αCDH2), with anti-β-actin (αβ-actin) as loading control. **d** WBs using NP69-anti-miR-34c stable cells and their control. Full-length blots are presented in Additional file 6: Figure S6. (E) WBs using NP460-anti-miR-34c stable cells and their control. ZO-1 was undetectable in NP460 cell lines. Full-length blots are presented in Additional file 7: Figure S7. **f** Relative SOX2 expression assessed by qRT-PCR in C666–1 cells treated with SB431542 (10 or 20 μM). The data are expressed as the mean ± SEM of at least three independent experiments. * *P* < 0.05; *** *P* < 0.001

SOX4 has been characterized as a master regulator of EMT [25, 27], notably by upregulating SOX2 [19–22], a well-known mediator of tumour initiation and cancer stem cell maintenance [72–74]. We therefore hypothesized that miR-34c could affect EMT via SOX4 and SOX2. First, SOX2 was confirmed to be highly expressed in C666–1 cells (low miR-34c; high SOX4) compared to NP69 and NP460 cells (high miR-34c; low SOX4) (Fig. 3a). NP69 cells stably expressing SOX4 had a significant increase in SOX2 expression (Additional file 3: Figure S3A), corroborating previous reports [19–22]. Moreover, downregulation of miR-34c in both NP69 and NP460 anti-miR-34c stable cell lines led to the significant upregulation of SOX2 (Fig. 3b and c). The overexpression of miR-34c in C666–1 correspondingly decreased SOX2 transcript levels (Additional file 3: Figure S3B).

The expression of well-known EMT markers were then investigated. NP69 anti-miR-34c stable cells overexpressed SNAI1 (Snail), ZEB1, and CDH2, while under-expressing CLDN1 (Claudin-1), ZO-1, and CDH1 (Fig. 3d). Similar results were observed in NP460 anti-miR-34c stable cells (Fig. 3e), supporting the role of miR-34c downregulation in the promotion of EMT in normal nasopharyngeal cell lines. C666–1 cells were not amenable to this gene expression analysis (ZEB1, CDH2, and CLDN1 are not expressed). However, TGFBR1 inhibition using SB431542 decreased SOX2 transcript expression in C666–1 cells (Fig. 3f). Taken together, the data show that high levels of TGFβ1 downregulate miR-34c, which directly leads to SOX4 overexpression and consequent SOX2 upregulation, promoting EMT in nasopharyngeal cells.

### TGFBR1 inhibition sensitizes C666–1 cells to cisplatin

**Fig. 4:**
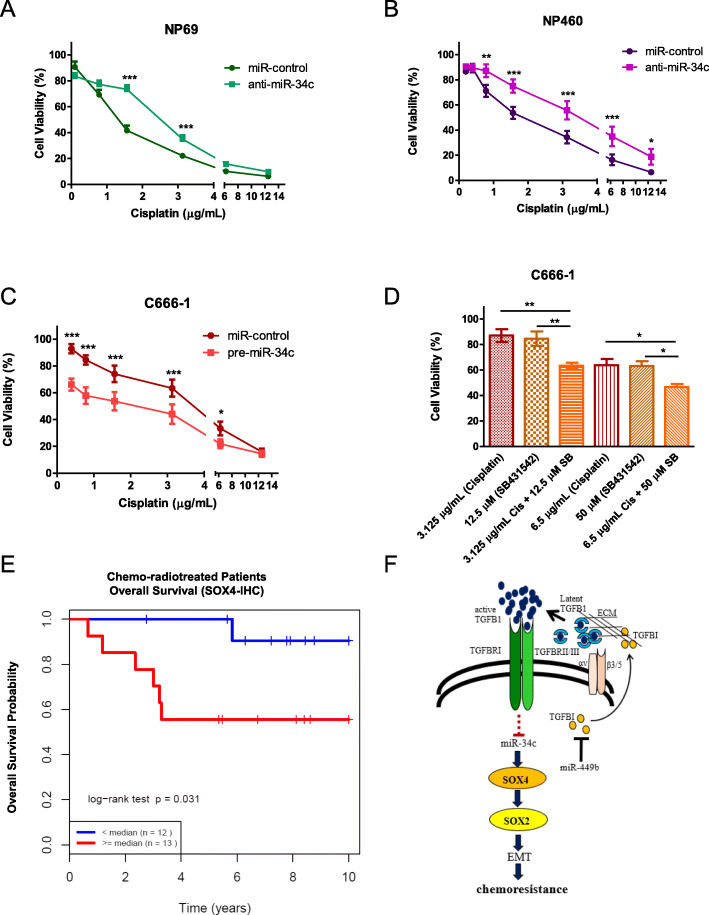
MiR-34c and the TGFβ pathway are involved in cisplatin sensitivity in NPC cells. **a** to **d** Cell viability was assessed 72 h after cisplatin treatment using the ATPlite assay. **a** Stable NP69-anti-miR-34c (or control) cells. **b** Stable NP460-anti-miR-34c (or control) cells. **c** Stable C666–1-pre-miR-34c (or control) cells. **d** C666–1 cells were treated simultaneously with combinations of cisplatin and SB431542 with varying doses. The data are represented as the mean ± SEM of at least three independent experiments. * *P* < 0.05; ** *P* < 0.01; *** *P* < 0.001. **e** Kaplan-Meier curve of OS based on low (<median) vs. high (>median) SOX4 expression (nuclear staining of tumour cells) using an anti-SOX4 polyclonal antibody in 25 NPC patients treated with chemoradiation (median follow-up time = 5 years). **f** Proposed model for the miR-449-TGFβ1-miR-34c-SOX4 pathway [12]. The red dotted line indicates that the mechanism remains unknown

Our group previously demonstrated that miR-449b overexpression was associated with EMT and cisplatin sensitivity in NPC [12], with EMT being a well-described mediator of chemoresistance [75]. In this current study, miR-34c was found to be downregulated by TGFβ1 (Fig. 1), leading to EMT. On this basis, the potential involvement of miR-34c in cisplatin resistance was examined. Downregulation of miR-34c using anti-miR-34c significantly increased resistance to cisplatin in NP69, NP460, and C666–1 stable cell lines (Fig. 4a and b, Additional file 3: Figure S3C). Conversely, overexpression of miR-34c using pre-miR-34c increased cisplatin sensitivity in NP69, NP460, and C666–1 stable cell lines (Additional file 3: Figure S3D and E, Fig. 4c). Additionally, SB431542 treatment had a cytotoxic effect on C666–1 cells in a dose-dependent manner in vitro (Additional file 3: Figure S3F). The combination of SB431542 and cisplatin had an additive effect on the cell death of C666–1 cells (Fig. 4d). Finally, IHC performed on NPC biopsy samples from patients treated with chemoradiation (*n* = 25) demonstrated that lower SOX4 nuclear immunostaining was associated with a superior 10-year OS compared to patients with high SOX4 immunostaining (*p* = 0.031; Fig. 4e, and Additional file 4: Figure S4). These data all support a role for the TGFβ1-miR34c-SOX4-SOX2 pathway in mediating cisplatin sensitivity in NPC.

In summary, miR-34c acts as a switch that controls EMT and chemoresistance in NPC. With TGFβ1 stimulation, miR-34c is repressed, directly leading to an increase in SOX4, which consequently upregulates SOX2, leading to EMT and cisplatin resistance in NPC (Fig. 4f).

## Discussion

This study revealed a novel role of miR-34c in EMT and chemoresistance in NPC. Downregulation of miR-34c in our cellular model, caused at least partially by miR-449b overexpression and consequent TGFβ1 activity, resulted in SOX4 and SOX2 overexpression, which triggered EMT and cisplatin resistance (Fig. 4f). Concordantly, miR-34c overexpression sensitized NPC cells to cisplatin—a phenotype corroborated in other cancer types [76–79].

Interestingly, miR-34c and miR-449b belong to the same miRNA family, as their seed sequences are highly similar (reviewed in [80]). Despite having potentially overlapping predicted targets however, as illustrated in this study, they do not function in the same manner in every context. Our data do demonstrate a similar effect wherein both miR-449b and miR-34c lead to the same cellular outcome: EMT and cisplatin resistance. Further experiments would be required to unravel the roles of the other members of the miR-34/449 family in NPC.

In NPC, miR-34c downregulation has been previously reported by several groups [11, 38, 39], but its mechanism of action has never been determined. This study elucidated a clear signaling pathway and provides data suggesting a myriad of other miR-34c effects. For example, our data demonstrated that miR-34c overexpression increased the expression of well-known pro-apoptotic genes, such as BAX [81] and PML [82]. Interestingly, the inhibition of PML nuclear bodies by the EBV protein EBNA1 has been described to contribute to tumorigenesis in NPC cells [83, 84]. MiR-34c has also been reported to suppress tumorigenesis through MET inhibition [38]. These and other miR-34c relationships remain to be further investigated in NPC.

Other miR-34 family members have been shown to be pro-apoptotic [44], with a liposome containing a miR-34a mimic (MRX34) being developed and evaluated clinically as a therapeutic agent [85]. Additionally, while miR-34a regulates SOX2 expression through PAI-1 [86], its overexpression reverts EMT, which suppresses invasion in NPC [53] and enhances docetaxel sensitivity in prostate cancer [87].

There has been increasing evidence supporting a primary role for TGFβ pathway activation in NPC [12, 53, 63, 65–67]. This current study demonstrated that miR-34c can be downregulated by TGFβ1, and that miR-449b overexpression can cause similar effects. Correspondingly, miR-449b upregulation and miR-34c downregulation were components of the four-miRNA prognostic signature for DM in NPC [11]. Cellular models mimicking these miRNA dysregulations display mesenchymal features and resistance to cisplatin, which are known contributors to disease recurrence and metastasis [12, 88, 89]. Furthermore, in C666–1 cells, TGFβ pathway inhibition produced a similar gene expression profile to transient miR-34c overexpression (i.e. NOTCH1, TGIF2, BAX, and PML), suggesting a close relationship between TGFβ1 and miR-34c pathways. The relationship between these pathways and chemoresistance should be a potential avenue of investigation for future translational studies.

## Conclusion

This study elucidates the novel role of miR-34c in EMT and cisplatin resistance. TGFβ1 negatively regulates miR-34c, which in turn increases the expression of SOX4 and SOX2, mediators of EMT triggering leading to cisplatin resistance (Fig. 4f). Correspondingly, miR-34c overexpression and TGFβ pathway inhibition leads to cisplatin sensitivity in NPC, highlighting a potential therapeutic strategy for this complex disease.

## Supplementary information

**Additional file 1: Figure S1**. (A) Relative miR-34c expression assessed by qRT-PCR in C666–1 cells treated with SB431542 (10 or 20 μM) for 72 h compared to untreated cells (UT). (B and C) Relative expression of putative miR-34c targets assessed by qRT-PCR in NP69, NP460, and C666–1 cells, normalized to C666–1 cells. (B) Genes that are highly expressed in C666–1 (NPC) cells vs. NP69 and NP460 (normal nasopharyngeal) cells. (C) Genes with no significant differences in expression between C666–1 cells and NP69/NP460. Note that MARCKS and PML expression were significant only between C666–1 and NP460 cells. The data are represented as the mean ± SEM of at least three independent experiments. * *P* < 0.05; ** *P* < 0.01; *** *P* < 0.001.

**Additional file 2: Figure S2**. (A) Relative expression of putative miR-34c target genes after transient transfection with pre-miR-34c (20 or 50 nM; 72 h after transfection) in C666–1 cells. (B) Relative expression of putative miR-34c target genes after SB431542 treatment (10 or 20 μM; 72 h) in C666–1 cells. (C to J) Relative expression of significantly dysregulated genes (ARID5A, BIK, LITAF, NFKBIA, SOX4, BAX, and PML) as assessed by qRT-PCR. (C) Gene expression after transient transfection with pre-miR-34c (20 or 50 nM) in NP69 cells. (D) Gene expression after transient transfection with pre-miR-34c (20 or 50 nM) in NP460 cells. (E) Gene expression of NP69-anti-miR-34c stable cells. (F) Gene expression of NP69-pre-miR-34c stable cells. (G) Gene expression of NP460-anti-miR-34c stable cells. (H) Gene expression of NP460-pre-miR-34c stable cells. (I) Gene expression of C666–1-anti-miR-34c stable cells. (J) Gene expression of C666–1-pre-miR-34c stable cells. The data are represented as the mean ± SEM of at least three independent experiments. * *P* < 0.05; ** *P* < 0.01; *** *P* < 0.001.

**Additional file 3: Figure S3**. (A) Relative SOX4 and SOX2 expression assessed by qRT-PCR in NP69 cells stably overexpressing SOX4. (B) Relative SOX2 expression assessed by qRT-PCR in C666–1 cells stably expressing pre-miR-34c. (C, D, and E) Cell viability was measured by ATPlite assay 72 h after cisplatin treatment: (C) Stable C666–1-anti-miR-34c (or control) cells. (D) Stable NP69-pre-miR-34c (or control) cells. (E) Stable NP460-pre-miR-34c (or control) cells. (F) Effect of SB431542 on C666–1 cell viability measured by ATPlite at 72 h. The data are represented as the mean ± SEM of at least three independent experiments. * *P* < 0.05; ** *P* < 0.01; *** *P* < 0.001.

**Additional file 4 Figure S4.** IHC was performed on NPC patient samples with an anti-SOX4 polyclonal antibody. Representative photomicrographs of SOX4 expression in the tumour nuclei of scores 0, 1, and 2 at 200X. No samples presented with a score of 3.

**Additional file 5: Figure S5.** Uncropped Western blots for Fig. 1c and f.

**Additional file 6: Figure S6**. Uncropped Western blots for Fig. 3d.

**Additional file 7: Figure S7**. Uncropped Western blots for Fig. 3e.

## Data Availability

The datasets used and/or analysed during the current study are available from the corresponding author on reasonable request.

## Abbreviations

3’UTR: 3′ untranslated region
CDDP: Cisplatin
CDH1: E-cadherin
CDH2: N-cadherin
CLDN1: Claudin-1
DM: Distant metastasis
DMEM: Dulbecco’s Modified Eagle Media
DRFS: Distant relapse-free survival
EBV: Epstein-Barr virus
EMT: Epithelial-to-mesenchymal transition
FBS: Fetal bovine serum
FFPE: Formalin-fixed and paraffin-embedded
IHC: Immunohistochemistry
MEM: Minimum Essential Media
miRNA: microRNA
NPC: Nasopharyngeal carcinoma
OS: Overall survival
qRT-PCR: quantitative real-time PCR
RT: Radiation therapy
SOX: SRY-related HMG-box
TGFβ1: Transforming growth factor beta 1
TGFBI: Transforming growth factor beta-induced
WCL: Whole Cell Lysate
WT: Wild type
ZEB1: Zinc finger E-box binding homeobox 1
