# Supplementary material for: MiR-34c downregulation leads to SOX4 overexpression and cisplatin resistance in nasopharyngeal carcinoma
Source: BMC Cancer. 2020 Jun 26;20:597. doi: 10.1186/s12885-020-07081-z (PMC7318489; doi:10.1186/s12885-020-07081-z)
Supplement: Supplementary file 1 — Additional file 1: Figure S1. (A) Relative miR-34c expression assessed by qRT-PCR in C666–1 cells treated with SB431542 (10 or 20 μM) for 72 h compared to untreated cells (UT). (B and C) Relative expression of putative miR-34c targets assessed by qRT-PCR in NP69, NP460, and C666–1 cells, normalized to C666–1 cells. (B) Genes that are highly expressed in C666–1 (NPC) cells vs. NP69 and NP460 (normal nasopharyngeal) cells. (C) Genes with no significant differences in expression between C666–1 cells and NP69/NP460. Note that MARCKS and PML expression were significant only between C666–1 and NP460 cells. The data are represented as the mean ± SEM of at least three independent experiments. * P < 0.05; ** P < 0.01; *** P < 0.001. [file 12885_2020_7081_MOESM1_ESM.docx]

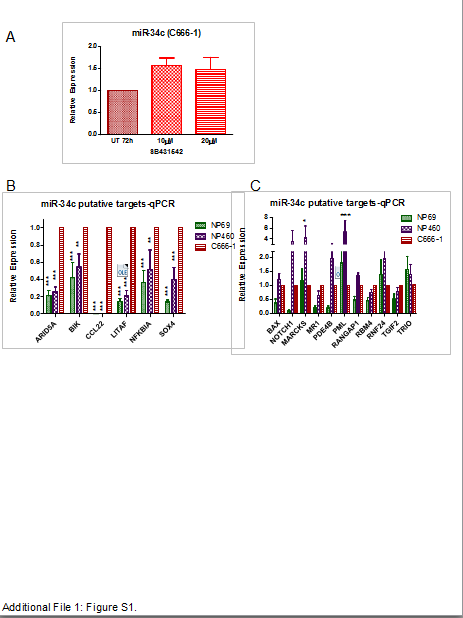


**Figure S1**. (A) Relative miR-34c expression assessed by qRT-PCR in C666-1 cells treated with SB431542 (10 or 20 µM) for 72 h compared to untreated cells (UT). (B and C) Relative expression of putative miR-34c targets assessed by qRT-PCR in NP69, NP460, and C666-1 cells, normalized to C666-1 cells. (B) Genes that are highly expressed in C666-1 (NPC) cells *vs.* NP69 and NP460 (normal nasopharyngeal) cells. (C) Genes with no significant differences in expression between C666-1 cells and NP69/NP460. Note that MARCKS and PML expression were significant only between C666-1 and NP460 cells. The data are represented as the mean ± SEM of at least three independent experiments.

* P<0.05; ** P<0.01; *** P<0.001.
