# Supplementary material for: MiR-34c downregulation leads to SOX4 overexpression and cisplatin resistance in nasopharyngeal carcinoma
Source: BMC Cancer. 2020 Jun 26;20:597. doi: 10.1186/s12885-020-07081-z (PMC7318489; doi:10.1186/s12885-020-07081-z)
Supplement: Supplementary file 2 — Additional file 2: Figure S2. (A) Relative expression of putative miR-34c target genes after transient transfection with pre-miR-34c (20 or 50 nM; 72 h after transfection) in C666–1 cells. (B) Relative expression of putative miR-34c target genes after SB431542 treatment (10 or 20 μM; 72 h) in C666–1 cells. (C to J) Relative expression of significantly dysregulated genes (ARID5A, BIK, LITAF, NFKBIA, SOX4, BAX, and PML) as assessed by qRT-PCR. (C) Gene expression after transient transfection with pre-miR-34c (20 or 50 nM) in NP69 cells. (D) Gene expression after transient transfection with pre-miR-34c (20 or 50 nM) in NP460 cells. (E) Gene expression of NP69-anti-miR-34c stable cells. (F) Gene expression of NP69-pre-miR-34c stable cells. (G) Gene expression of NP460-anti-miR-34c stable cells. (H) Gene expression of NP460-pre-miR-34c stable cells. (I) Gene expression of C666–1-anti-miR-34c stable cells. (J) Gene expression of C666–1-pre-miR-34c stable cells. The data are represented as the mean ± SEM of at least three independent experiments. * P < 0.05; ** P < 0.01; *** P < 0.001. [file 12885_2020_7081_MOESM2_ESM.docx]

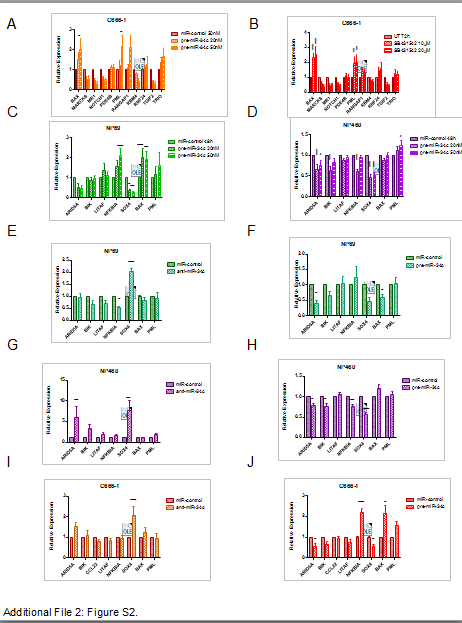


**Figure S2**. (A) Relative expression of putative miR-34c target genes after transient transfection with pre-miR-34c (20 or 50 nM; 72 h after transfection) in C666-1 cells. (B) Relative expression of putative miR-34c target genes after SB431542 treatment (10 or 20 µM; 72 h) in C666-1 cells. (C to J) Relative expression of significantly dysregulated genes (ARID5A, BIK, LITAF, NFKBIA, SOX4, BAX, and PML) as assessed by qRT-PCR. (C) Gene expression after transient transfection with pre-miR-34c (20 or 50 nM) in NP69 cells. (D) Gene expression after transient transfection with pre-miR-34c (20 or 50 nM) in NP460 cells. (E) Gene expression of NP69-anti-miR-34c stable cells. (F) Gene expression of NP69-pre-miR-34c stable cells. (G) Gene expression of NP460-anti-miR-34c stable cells. (H) Gene expression of NP460-pre-miR-34c stable cells. (I) Gene expression of C666-1-anti-miR-34c stable cells. (J) Gene expression of C666-1-pre-miR-34c stable cells. The data are represented as the mean ± SEM of at least three independent experiments.

* P<0.05; ** P<0.01; *** P<0.001.
