# Supplementary material for: MiR-34c downregulation leads to SOX4 overexpression and cisplatin resistance in nasopharyngeal carcinoma
Source: BMC Cancer. 2020 Jun 26;20:597. doi: 10.1186/s12885-020-07081-z (PMC7318489; doi:10.1186/s12885-020-07081-z)
Supplement: Supplementary file 3 — Additional file 3: Figure S3. (A) Relative SOX4 and SOX2 expression assessed by qRT-PCR in NP69 cells stably overexpressing SOX4. (B) Relative SOX2 expression assessed by qRT-PCR in C666–1 cells stably expressing pre-miR-34c. (C, D, and E) Cell viability was measured by ATPlite assay 72 h after cisplatin treatment: (C) Stable C666–1-anti-miR-34c (or control) cells. (D) Stable NP69-pre-miR-34c (or control) cells. (E) Stable NP460-pre-miR-34c (or control) cells. (F) Effect of SB431542 on C666–1 cell viability measured by ATPlite at 72 h. The data are represented as the mean ± SEM of at least three independent experiments. * P < 0.05; ** P < 0.01; *** P < 0.001. [file 12885_2020_7081_MOESM3_ESM.docx]

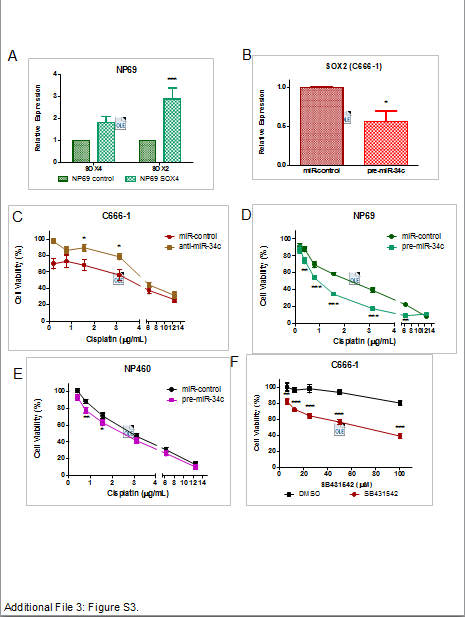


**Figure S3**. (A) Relative SOX4 and SOX2 expression assessed by qRT-PCR in NP69 cells stably overexpressing SOX4. (B) Relative SOX2 expression assessed by qRT-PCR in C666-1 cells stably expressing pre-miR-34c. (C, D, and E) Cell viability was measured by ATPlite assay 72 h after cisplatin treatment: (C) Stable C666-1-anti-miR-34c (or control) cells. (D) Stable NP69-pre-miR-34c (or control) cells. (E) Stable NP460-pre-miR-34c (or control) cells. (F) Effect of SB431542 on C666-1 cell viability measured by ATPlite at 72 h. The data are represented as the mean ± SEM of at least three independent experiments. * P<0.05; ** P<0.01; *** P<0.001.
