# Supplementary material for: MiR-34c downregulation leads to SOX4 overexpression and cisplatin resistance in nasopharyngeal carcinoma
Source: BMC Cancer. 2020 Jun 26;20:597. doi: 10.1186/s12885-020-07081-z (PMC7318489; doi:10.1186/s12885-020-07081-z)
Supplement: Supplementary file 4 — Additional file 4 Figure S4. IHC was performed on NPC patient samples with an anti-SOX4 polyclonal antibody. Representative photomicrographs of SOX4 expression in the tumour nuclei of scores 0, 1, and 2 at 200X. No samples presented with a score of 3. [file 12885_2020_7081_MOESM4_ESM.docx]

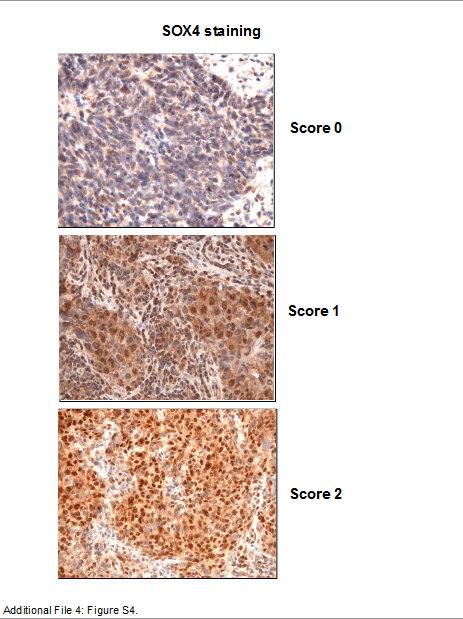


**Figure S4.** IHC was performed on NPC patient samples with an anti-SOX4 polyclonal antibody. Representative photomicrographs of SOX4 expression in the tumour nuclei of scores 0, 1, and 2 at 200X. No samples presented with a score of 3.
