# Supplementary figures and images for: MiR-34c downregulation leads to SOX4 overexpression and cisplatin resistance in nasopharyngeal carcinoma
Source: BMC Cancer. 2020 Jun 26;20:597. doi: 10.1186/s12885-020-07081-z (PMC7318489; doi:10.1186/s12885-020-07081-z)

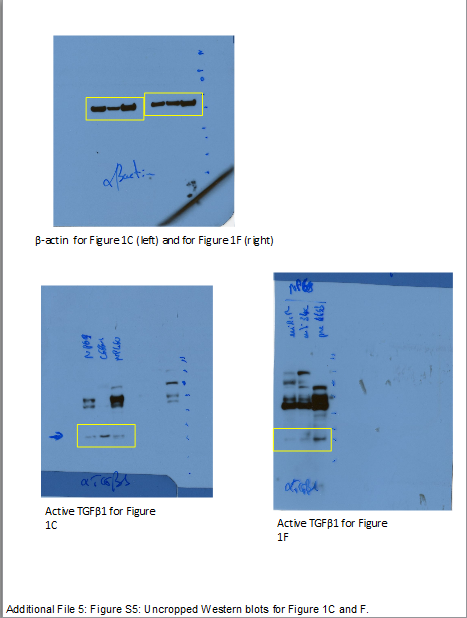


Figure S5. Uncropped Western blots for Figure 1C and F.

Supplement: Supplementary file 5 — Additional file 5: Figure S5. Uncropped Western blots for Fig. 1c and f. [file 12885_2020_7081_MOESM5_ESM.docx]

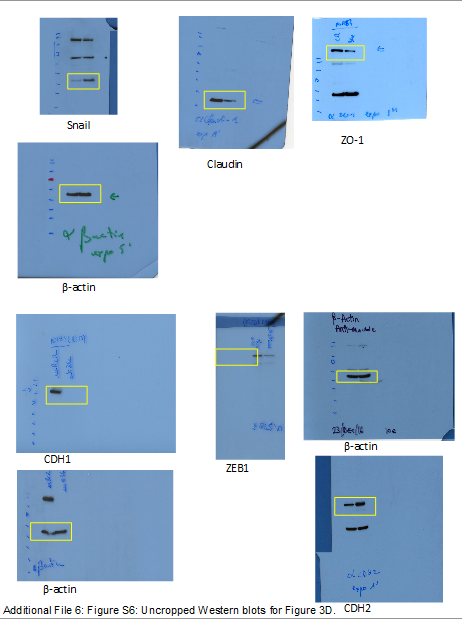


Figure S6. Uncropped Western blots for Figure 3D.

Supplement: Supplementary file 6 — Additional file 6: Figure S6. Uncropped Western blots for Fig. 3d. [file 12885_2020_7081_MOESM6_ESM.docx]

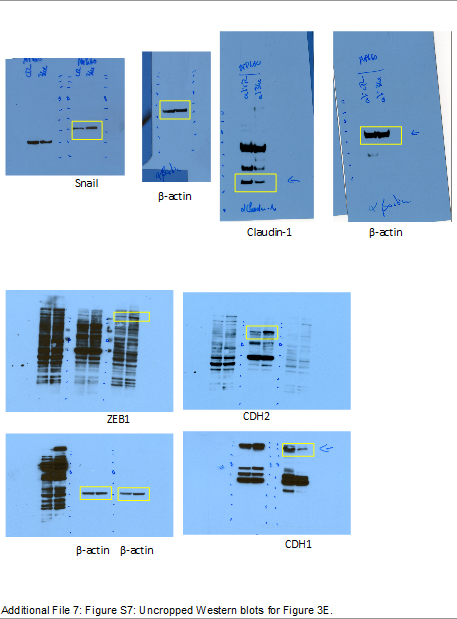


Figure S7. Uncropped Western blots for Figure 3E.

Supplement: Supplementary file 7 — Additional file 7: Figure S7. Uncropped Western blots for Fig. 3e. [file 12885_2020_7081_MOESM7_ESM.docx]
